# Anti-cancer Therapy Leads to Increased Cardiovascular Susceptibility to COVID-19

**DOI:** 10.3389/fcvm.2021.634291

**Published:** 2021-04-23

**Authors:** Caroline Lozahic, Helen Maddock, Hardip Sandhu

**Affiliations:** Faculty Research Centre for Sport, Exercise and Life Sciences, Faculty of Health and Life Sciences, Coventry University, Coventry, United Kingdom

**Keywords:** SARS-CoV-2, COVID-19, ACE2, cytokine storm, anti-cancer drug-induced cardiac injury

## Abstract

Anti-cancer treatment regimens can lead to both acute- and long-term myocardial injury due to off-target effects. Besides, cancer patients and survivors are severely immunocompromised due to the harsh effect of anti-cancer therapy targeting the bone marrow cells. Cancer patients and survivors can therefore be potentially extremely clinically vulnerable and at risk from infectious diseases. The recent global outbreak of the novel coronavirus severe acute respiratory syndrome coronavirus 2 (SARS-CoV-2) and its infection called coronavirus disease 2019 (COVID-19) has rapidly become a worldwide health emergency, and on March 11, 2020, COVID-19 was declared a global pandemic by the World Health Organization (WHO). A high fatality rate has been reported in COVID-19 patients suffering from underlying cardiovascular diseases. This highlights the critical and crucial aspect of monitoring cancer patients and survivors for potential cardiovascular complications during this unprecedented health crisis involving the progressive worldwide spread of COVID-19. COVID-19 is primarily a respiratory disease; however, COVID-19 has shown cardiac injury symptoms similar to the cardiotoxicity associated with anti-cancer therapy, including arrhythmia, myocardial injury and infarction, and heart failure. Due to the significant prevalence of micro- and macro-emboli and damaged vessels, clinicians worldwide have begun to consider whether COVID-19 may in fact be as much a vascular disease as a respiratory disease. However, the underlying mechanisms and pathways facilitating the COVID-19-induced cardiac injury in cancer and non-cancer patients remain unclear. Investigations into whether COVID-19 cardiac injury and anti-cancer drug-induced cardiac injury in cancer patients and survivors might synergistically increase the cardiovascular complications and comorbidity risk through a “two-hit” model are needed. Identification of cardiac injury mechanisms and pathways associated with COVID-19 development overlapping with anti-cancer therapy could help clinicians to allow a more optimized prognosis and treatment of cancer survivors suffering from COVID-19. The following review will focus on summarizing the harmful cardiovascular risk of COVID-19 in cancer patients and survivors treated with an anti-cancer drug. This review will improve the knowledge of COVID-19 impact in the field of cardio-oncology and potentially improve the outcome of patients.

## Introduction

For the past 40 years, the cancer survival rate has increased considerably due to the improvement in cancer diagnosis and treatment (1, 2). Unfortunately, anti-cancer drug therapy of cancer patients can lead to serious cardiac injury adverse effects, such as hypertension, arrhythmia, stroke, and heart failure (3). These harmful anti-cancer drug-mediated cardiac injury adverse effects are difficult to diagnose and prevent and can appear years after the anti-cancer treatment is completed. Regrettably, long-term anti-cancer drug-mediated cardiac injury can lead to increased mortality of cancer survivors (4). Anti-cancer therapy is very harsh and can adversely affect the bone marrow cells, which can lead to a severe immune deficiency in cancer patients and survivors (5). In particular, a delay of the adaptive response with antibodies is observed in cancer patients and survivors, and this can lead to an increase in the susceptibility to develop severe infections caused by common viruses like influenza (6, 7).

A novel coronavirus named SARS-CoV-2 causing COVID-19 appeared in Wuhan, China, toward the end of 2019. Within a few months, SARS-CoV-2 spread all over the world and was declared as a global pandemic by WHO on the March 11, 2020. Initial studies showed that COVID-19 mainly targeted and damage the respiratory system (8–11); however, recent studies have reported that COVID-19 can also induce cardiovascular complications, such as dysrhythmias, venous thromboembolic events, myocarditis, myocardial injury, acute myocardial infarction, and heart failure (12).

The myocardial injury associated with COVID-19 could harm cancer survivors, who are already at high risk of suffering from an anti-cancer therapy-induced cardiac injury (13). The possible relation and overlap between the myocardial injury mechanism of COVID-19 and the anti-cancer therapy-induced cardiac injury is a major cause of concern in the clinic. In this review, we will identify cardiac injury mechanisms and pathways during COVID-19 development and the possible comorbidity risk through a “two-hit” model cancer patients and survivors are facing.

## Anti-cancer Therapy and Cardiac Injury

### Anti-cancer Drugs Associated With Cardiac Injury

While the anti-cancer therapy options available to cancer patients have improved over the past decades, a rise in cardiac adverse effects as a result of anti-cancer therapy has emerged as a considerable cause for concern in cancer survivors. Various types of chemotherapeutic anti-cancer drugs can induce cardiac injury (14). Some of the most potent cardiotoxic anti-cancer drugs belong to the anthracycline group, such as doxorubicin (3). Anthracyclines can induce cardiac injury through different intracellular mechanisms including oxidative stress (15), mitochondrial dysfunction, reactive oxygen species production (16), apoptosis (17), and myofibril damage (18). Other chemotherapy drugs responsible for cardiac complications include taxoids, antimetabolites, immune checkpoint inhibitors (ICIs), alkylating agents, and tyrosine kinase inhibitors (19, 20). The antimetabolite 5-fluorouracil can interfere with the nucleic acid function (21), and 5-fluorouracil treatment can induce arrhythmia, silent myocardial ischemia, and congestive heart failure in cancer patients (3). Therapy with ICIs has been associated with atherosclerosis, venous thromboembolism, vasculitis, Takotsubo syndrome, myocarditis, and hypertension (22–24). The alkylating agent cyclophosphamide has both cardiotoxic and immunosuppressive adverse effects (25), and cyclophosphamide treatment can lead to arrhythmia, cardiac tamponade, and congestive heart failure (3, 26). The tyrosine kinase inhibitor sunitinib inhibits ribosomal S6 kinase activity to induce cellular apoptosis (27), and sunitinib therapy can induce cardiomyocyte death and hypertrophy, which can lead to hypertension or, in the severe case, to congestive heart failure (27).

### Assessment of Cardiac Injury

Anti-cancer therapy-induced cardiac injury can be assessed by imaging techniques and various circulating myocardial injury biomarkers. Doppler echocardiography and echocardiographic left ventricular ejection fraction (LVEF) are non-invasive techniques used in the clinic to detect myocardial injury (28). Cardiac injury can also be monitored through assessment of relevant circulating biomarkers levels, such as cardiac troponin I (cTnI) and B-type natriuretic peptide (BNP) (29). Cardiac damages lead to an increase in cTnI, while the rise of left ventricle wall stress leads to increased BNP levels (29). The “gold standard” in the clinic to determine the onset and stage of cardiac injury is a combination of LVEF assessment and determining the circulating levels of cTnI and BNP.

### Anti-cancer Therapy Can Lead to Immunocompromised Status in Cancer Patients

Cancer patients treated with anti-cancer drugs are at an increased risk of developing infections and have fatal outcomes as a result of immunodeficiency, due to the harsh effect of anti-cancer therapy on the bone marrow cells (30). Cancer survivors tend to have more frequently influenza infections and acute respiratory infections than the general population (31). Moreover, childhood cancer survivors are more likely to develop acute respiratory disease after influenza infection due to their weak immunity (7). Further to this, pre-existing cardiovascular diseases can aggravate in high-risk cancer survivors (32). The infection-related complications observed in cancer survivors highlight that they are at high risk from the new COVID-19 pandemic.

## Novel Global Pandemic COVID-19

### SARS-CoV-2 Transmission

The novel zoonotic RNA virus SARS-CoV-2 leading to COVID-19 is from a large family of enveloped single-stranded zoonotic RNA viruses (9). WHO has reported over 113 million COVID-19 cases and over 2.5 million COVID-19-related deaths worldwide (by March 1, 2021). SARS-CoV-2 is transmitted human-to-human by indirect or direct contact through mucus membranes (i.e., mouth, eyes, or nose). Transmission is primarily mediated through airborne microparticles encapsulating the SARS-CoV-2 viral inoculum being inhaled in the nasal cavity, followed by aspiration of the SARS-CoV-2 microparticles into the lung, where SARS-CoV-2 then triggers the viral infection (33).

### COVID-19 Symptoms

SARS-CoV-2 infection can induce systematic and respiratory disorders (34). The most common symptoms of COVID-19 are high fever, consistent dry cough, and loss of smell and taste sense (10). Several COVID-19 patients showed symptoms of viral pneumonia, such as sore throat, fatigue, and myalgia (11). The most serious cases of SARS-CoV-2 infections have fatal outcomes. The period from the onset of symptoms to death ranges from 6 to 41 days, depending on the vulnerability of the patient (35).

COVID-19 was first considered as a respiratory disease, due to the acute respiratory distress syndrome (ARDS). Indeed, in critical COVID-19 cases, patients suffer from dyspnoea, RNAaemia (i.e., detection of SARS-CoV-2 RNA in blood serum), and hypoxemia, and ground-glass opacities have been observed in sub-pleural regions of both of their lungs (36, 37). However, recent studies have shown that COVID-19 can cause inflammation-associated complications in other systems, such as the cardiovascular, cerebral, and gastrointestinal systems (10).

### COVID-19 Risk Factors

**Figure 1:**
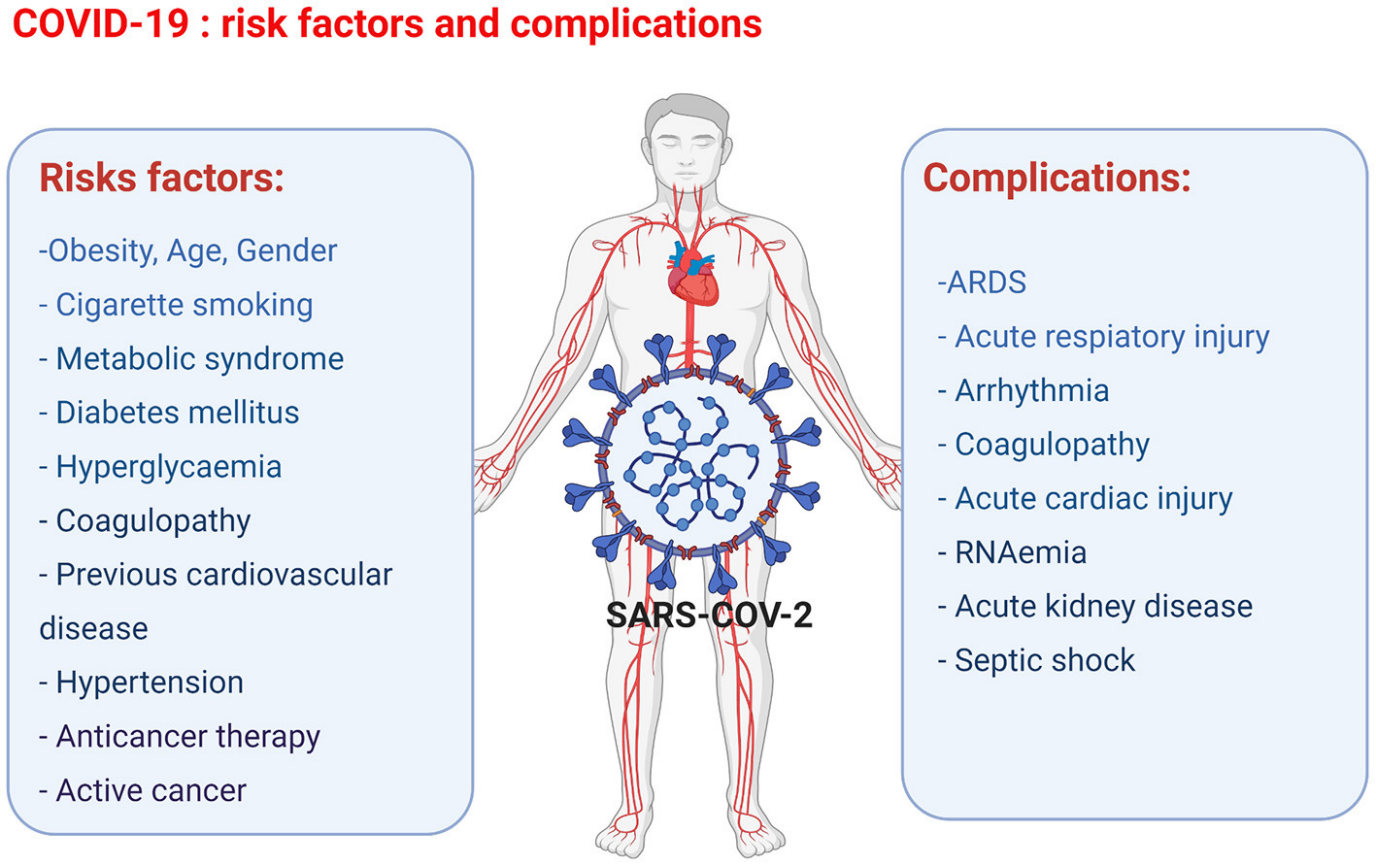
Overview of the COVID-19 risk factors and complications observed in COVID-19 patients leading to cardiac injury (figure created with Biorender.com).

The key prognostic risk factors in patients with COVID-19 include obesity, metabolic syndrome, diabetes mellitus, hyperglycaemia, coagulopathy, cigarette smoking, previous cardiovascular diseases, hypertension, previous cardiotoxic anticancer therapies, and active cancer. Furthermore, in cancer patients infected with COVID-19, the additional risk factors include various active anti-cancer treatments, such as chemotherapy, radiotherapy, and bone marrow or stem cell transplants (24). The most consistent predictors of poor outcomes of COVID-19 are age and gender (Figure 1). A higher incidence of fatalities is observed in the elderly COVID-19 patients compared to younger patients. The exact mechanisms involved are yet to be discovered; however, the increased prevalence of frailty and cardiovascular disease in the elderly population is thought to be due to pre-existing endothelial dysfunction and loss of endogenous cardioprotective mechanisms (38). Gender also plays an important factor in the clinical outcome following the contraction of COVID-19. Regardless of their age, women are less susceptible to severe infection outcomes, and fewer women are dying of COVID-19-related complications than age-matched males. The gender-associated difference in COVID-19 outcome is thought to be linked to two key mediators of viral attachment to cell membranes, namely, the angiotensin-converting enzyme (ACE) 2 receptor (ACE2) for the spike protein of coronaviruses, and the type 2 transmembrane serine protease (TMPRSS2), which cleaves the spike protein and thereby facilitates the attachment and fusion of the virus to cell membranes (39, 40). In particular, the ACE and ACE2 expression levels and the resulting ACE/ACE2 activity ratio are important factors for COVID-19 infection outcome. ACE has pro-inflammatory and pro-oxidant effects, whereas ACE2 has anti-inflammatory and antioxidant effects (41, 42). The ACE/ACE2 activity ratio has shown to be lower in serum samples of females compared to male serum samples, and the lower ACE/ACE2 activity ratio could be one of the factors that protect female COVID-19 patients from developing severe complications (42). In older COVID-19 patients, the worse outcome has been attributed to the presence of lower ACE2 levels compared to younger patients and thus the subsequent upregulation of pro-inflammatory pathways through angiotensin II (43).

### COVID-19 Mechanism of Action

#### SARS-CoV-2 Transmission and Replication

**Figure 2:**
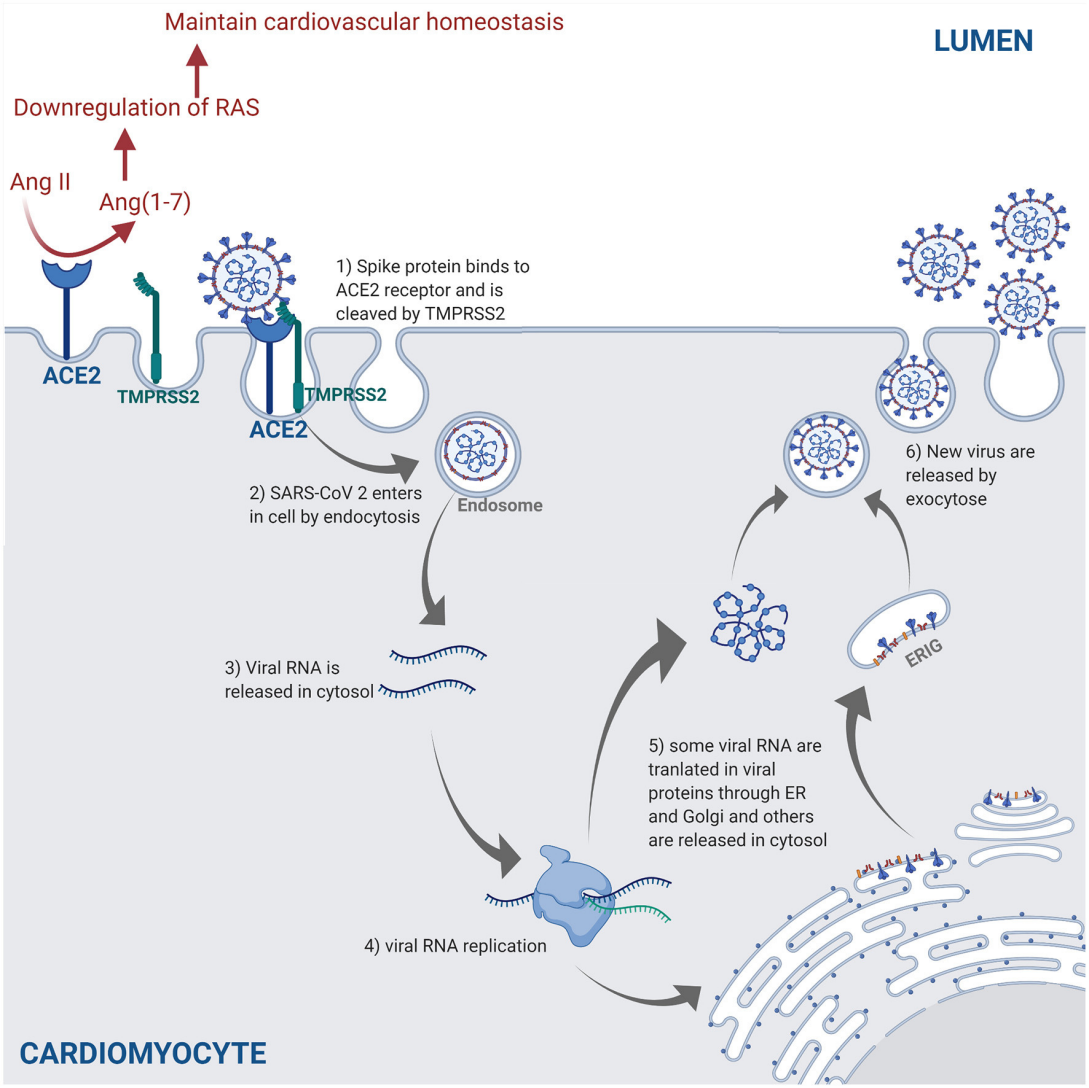
The SARS-CoV-2 pathway in the host cells. The spike protein of the virus binds to the cellular receptor angiotensin-converting enzyme 2 (ACE2) and is cleaved by the type 2 transmembrane serine protease (TMPRSS2). This allows entry into the host cell by endocytosis. Following the entry of SARS-CoV-2, viral RNA is released into the cytoplasm and replicated. Viral RNA is translated by the endoplasmic reticulum (ER) by Golgi complex and form the endoplasmic reticulum–Golgi intermediate compartment (ERIG). Viral RNA enters into ERIG by budding to form novel virions. These virions are released from the infected cells by exocytosis. The ACE2 receptor is a negative regulator of the renin angiotensin system (RAS) and converts angiotensin II (Ang II) into angiotensin 1–7 [Ang(1–7)]. The downregulation of the RAS allows to maintain the cardiovascular homeostasis (figure created with Biorender.com).

SARS-CoV-2 is transmitted through the surface mucus membranes in the host human cells, where the viral surface spike protein binds to the cellular entry receptor ACE2 facilitating viral entry into host cells (44). The cellular serine protease TMPRSS2 cleaves the SARS-CoV-2 spike protein, thus allowing the attachment and fusion of SARS-CoV-2 virus to the host cell membrane and release of viral RNA in the host cell cytosol (40). The viral RNA is translated in the endoplasmic reticulum by the Golgi complex to produce more virions contained in double membrane vesicles, which are released in the extracellular matrix by exocytosis (45, 46) (Figure 2).

#### ACE2 Receptors Involved in Cardiac Function

ACE2 is highly expressed in lung, heart, and kidneys, and is a key negative regulator of the renin angiotensin system (RAS) through angiotensin II expression adjustments (47). SARS-CoV-2 downregulates ACE2 expression by binding to ACE2 receptors (47). In a murine model, the decrease in ACE2 promoted an increase of angiotensin II level in alveolar cells, which causes an increase of pulmonary vascular permeability leading to pulmonary oedema and lung dysfunction (48). Another study demonstrated that the loss of ACE2 promoted the upregulation of hypoxia-inducible gene (i.e., BNIP3 and PAI-1) expression in a murine model (49). All in all, the increase of vascular permeability, hypoxia, and hyper-inflammation caused by COVID-19 leads to ARDS, which is characterized by pulmonary oedema (50).

The ACE2 receptors are also expressed in vascular endothelium cells (51) and are key regulators of cardiovascular homeostasis (52). Knockout of the ACE2 gene expression in murine hearts leads to an increase in angiotensin II levels and severe diminution of cardiac contractility when compared to wild-type mice, thus indicating that ACE2 receptors are essential regulators of cardiac function and ACE2 loss induces myocardial injury (49).

#### SARS-CoV-2 Triggered Immune Response

**Figure 3:**
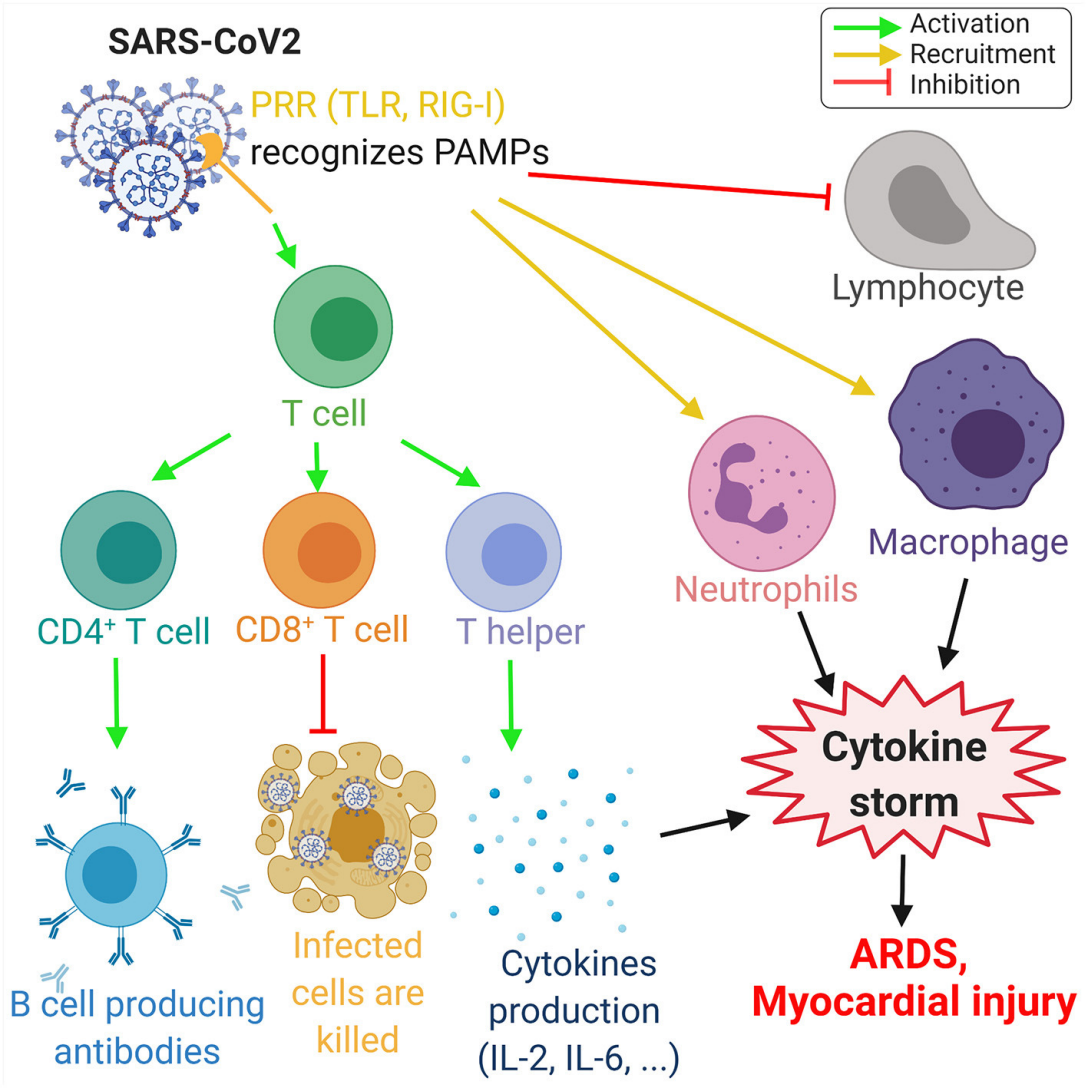
Overview of the immune response to COVID-19. The proteins on SARS-CoV-2 membrane are recognized as pathogen-associated molecular pattern by the pattern recognition receptors (PRRs), such as Toll-like receptors (TLR) and retinoic acid-inducible gene-I (RIG-I)-like receptors. This recognition leads to the activation of T-cells, which differentiate in CD4^+^ T-cells, CD8^+^ T-cells, and T-helper cells that induce, respectively, the production of antibodies by B-cells, the deaths of infected cells, and the cytokine production, such as interleukins IL-2 and IL-6. Moreover, SARS-CoV-2 invasion leads to the recruitment of neutrophils and macrophage and induce a lymphopenia. The increase of neutrophils and macrophages associated with the cytokine production leads to a hyperinflammation called the cytokine storm. This cytokine storm leads to acute respiratory distress syndrome (ARDS) and myocardial damage. Green arrows refer to activating effects, orange arrows refer to recruitment effects, and the red lines refer to inhibiting effects (figure created with Biorender.com).

A viral infection is followed by the innate and adaptive responses of the immune system. In the innate response, the viral RNA is considered as a pathogen-associated molecular pattern and is detected by the pattern recognition receptors. Viral RNA is detected by the Toll-like receptors (TLR) in the endosomes, and retinoic acid-inducible gene-I (RIG-I)-like receptors detect the viral RNA in the cytoplasm (53). This innate response is followed by the adaptive immune response by T-cells, which play a key role in viral infection by regulating the balance between the risk of autoimmunity and overactive inflammation. CD4^+^ T-cells promote the production of antibodies specific to the virus by activating T-dependent B-cells. In addition, T-helper cells produce pro-inflammatory cytokines via the nuclear factor kappa-light-chain-enhancer (NF-κβ) signaling pathway and regulatory T-cells maintain the homeostasis of the immune system. Finally, the cytotoxic CD8^+^ T-cells exterminate the infected cells through antigen recognition (53–55) (Figure 3).

In patients with COVID-19, several studies have highlighted the increase of neutrophils, leukocytes, and lymphopenia. The neutrophil–lymphocyte ratio is a marker of systemic inflammation due to an infection and is increased during COVID-19 (55). In a cohort study including 452 patients in Wuhan, China, an increase of interleukins IL-2, IL-6, IL-8, IL-10, and NF-κβ was observed in COVID-19-infected patients (55). IL-6 levels are increased during infection and IL-6 is a significant modulator of the cytokine storm. Moreover, there is a significant increase in the IL-6 levels in severe cases of COVID-19, when compared to mild COVID-19 cases (55, 56). A correlation between the severity of COVID-19 and the increase of pro-inflammatory cytokines have been observed in COVID-19 patients (57). COVID-19 damages specific tissue, which can aggravate the cytokine production, leading to the development of a cytokine storm, characterized by an excessive production of pro-inflammatory cytokines and the recruitment of macrophages and granulocytes. The cytokine storm can lead to more damage in lung tissue, ARDS (58), and myocardial injury (59).

### COVID-19 and Cardiac Injury

Cardiac complications in patients infected by SARS-CoV-2 have been reported, particularly in severe COVID-19 cases (60, 61). Thrombotic complications, such as microvascular thrombosis, venous thromboembolic disease, and stroke, have been identified in severe COVID-19 cases (62). The mechanism of action is thought to be SARS-CoV-2 invading ACE2 receptors expressed on the surface of endothelial cells (63). The subsequent endothelial inflammation process in COVID-19 patients induces a dysfunction of the endothelial homeostasis, and this can lead to severe and life-threatening (micro)thrombotic complications, such as pulmonary embolism, deep vein thrombosis, and stroke (64, 65). Further to this, ACE2 receptors are highly expressed in the heart and can lead to ACE2-dependent myocardial infarction (66). COVID-19 has also been shown to cause cardiac injury by directly targeting the cardiac cells (67, 68).

In a cohort of 416 COVID-19 patients, cardiac injury was determined by the assessment of serum cardiac markers levels, including high-sensitivity troponin I, and by measurement of abnormalities in electrocardiography readings. All cardiac injury trials in these COVID-19 patients were similar to those observed during myocardial ischemia. This cohort study showed a very high mortality rate of 51% among COVID-19 patients with cardiac injury complications. The study compared biomarkers levels and radiographic findings in 82 COVID-19 patients with cardiac injury with 334 COVID-19 patients without cardiac injury. According to their comparison, COVID-19 patients with cardiac injury had a significantly higher mortality risk than COVID-19 patients without cardiac injury (67). In another cohort study, 7.2% of the 138 hospitalized COVID-19 patients developed an acute cardiac injury after being infected by SARS-CoV-2, and these patients represented 22.2% of the COVID-19 patients admitted to the intensive care unit (ICU). The results of this study suggest that the manifestation of acute cardiac injury correlates to an imparted outcome in COVID-19 patients (69).

The cardiac injury mechanisms of action involved during COVID-19 infection remain unclear; however, it could be due to altered expression of ACE2 receptors and involvement of the cytokine storm (70, 71). Additionally, the myocardial injury could be caused by cardiac stress induced by hypoxia from ARDS (72). In two independent single-patient clinical case reports from China, the individual patients infected by SARS-CoV-2 were hospitalized and were suffering from both pneumonia and cardiac symptoms. The COVID-19 patients had elevated troponin T level, abnormal echocardiography, decreased LVEF, and elevated IL-6 levels, which could indicate a possible ongoing cytokine storm. The prognosis of clinicians was that the patients were suffering from fulminant myocarditis induced by SARS-CoV-2 (73, 74). Other studies have suggested that myocarditis represents 7% of deaths due to COVID-19 complications (75). However, most of these reports are based on assumptions and are not based on confirmed myocarditis diagnoses (76).

Studies have shown that COVID-19 patients suffering from severe complications are affected by arrhythmia (69, 77). Indeed, viral infections like COVID-19 are associated with myocardial inflammation, which can lead to cardiac arrhythmia (70). Other studies have observed an increase of D-dimer levels and fibrin degradation products in COVID-19 patients, and these features are known as indicators of disseminated intravascular coagulation and pulmonary embolism (70, 78). A report based on 106 pulmonary CT angiograms from COVID-19 patients showed that 30% of the patients had acute pulmonary with high D-dimer threshold and argued that this could be due to an increase in the blood coagulation level following the cytokine storm (79).

## COVID-19 and Cancer Patients and Survivors

### Increased COVID-19 Complications and Comorbidity in Cancer Patients and Survivors

Cancer patients have an estimated two-fold increased risk of contracting SARS-CoV-2 compared to the general population (80). In a cohort study of 1,590 Chinese patients suffering from COVID-19, it was noted that 1.13% of them were cancer patients/survivors, which is higher than the overall 0.29% cancer incidence in China. Among the 18 cancer survivors, 4 of them had completed chemotherapy or surgery the past month, 12 were cancer survivors in routine follow-up, and 2 did not report their cancer history. This study showed that COVID-19 patients with recently completed anti-cancer therapy were those experiencing the most severe COVID-19 complications, and some of these patients died before the onset of COVID-19 treatment. Therefore, recent completion of anti-cancer therapy seems to be an important risk factor for developing severe complications during COVID-19 infection (81).

In another study from Wuhan, China, 28 cancer patients with COVID-19 were monitored closely for complications: 54% of them developed severe complication, 21% were admitted to ICU, 38% were considered to be in a life-threatening condition, and 28% died as a result of their complication. The cancer survivors started with developing the most common symptoms of COVID-19 consisting of a high fever and a consistent cough; however, the COVID-19 complications escalated quickly and they also developed anamia and hypoproteinaemia, leading to a reduction of immunocompetence and increasing the risk of developing respiratory complications. The last two symptoms, anamia and hypoproteinaemia, are not common in COVID-19 patients without any anti-cancer therapy history. COVID-19 patients with a previous anti-cancer therapy history seem to develop dyspnoea at an earlier stage compared to COVID-19 patients without previous cancer history. This study strongly indicates that cancer patients and survivors might have a higher risk of developing viral infection with severe complications due to their immunocompromised status (82). Please refer to Table 1 for an overview of comorbidities and complications observed in COVID-19 patients suffering from cardiac injury identified in the studies included in this review.

**Table 1:** Comorbidities and complications in COVID-19 patients suffering from cardiac injury identified in the studies included in this review.

**References**	**COVID-19 patients (ICU admission %)**	**Male (%)**	**Age (median)**	**Comorbidities and cancer type (if specified)**	**COVID-19 complications**
Huang et al. (10)	41 (32% ICU)	73.00%	49	Diabetes (20%), Hypertension (15%), Cardiovascular diseases (15%), Chronic obstructive pulmonary disease (2%), Malignancy (2%), Chronic liver disease (2%).	ARDS (29%), RNAaemia (15%), Acute cardiac injury (12%), Secondary infection (10%), Acute kidney injury (7%), Shock (7%).
Qin et al. (55)	452	52.00%	58	Hypertension (29.5%), Tuberculosis (19.7%), Diabetes (16.4%), Cardiovascular disease (5.9%), Malignant tumor (3.1%), Chronic obstructive pulmonary disease (2.6%), Cerebrovascular disease (2.4%), Chronic kidney disease (2.2%), Chronic liver disease (1.3%).	–
Wichemann et al. (62)	12 (all dead)	75.00%	73	Coronary heart disease (50%), Arterial hypertension (25%), Chronic obstructive pulmonary disease (25%), Obesity (25%), Diabetes (25%), Bronchial asthma (25%), Nicotine abuse (16.7%), Atrial fibrillation (16.7%), Chronic kidney disease (16.7%), Parkinson disease (16.7%), Peripheral artery disease (8.3%), Granulomatous pneumopathy (8.3%), Dementia (8.3%), Epilepsy (8.3%), Trisomy 21 (8.3%), Non–small cell lung cancer (8.3%), Ulcerative colitis (8.3%).	Deep venous thrombosis (58%), pulmonary embolism (33%).
Shi et al. (67)	416	49.30%	64	Hypertension (30.5%), Diabetes (14.4%), Coronary heart disease (10.6%), Cerebrovascular disease (5.3%), Chronic heart failure (4.1%), Chronic renal failure (3.4%), Chronic obstructive pulmonary disease (2.9%), Cancer (2.2%), Hepatitis B infection (1.0%).	ARDS (23.3%), Electrolyte disturbance (7.2%), Hypoproteinemia (6.5%), Anamia (3.1%), Coagulation disorders (2.9%), Acute kidney injury (1.9%).
Han et al. (68)	273 (5.5% ICU)	35.53%	58.95	–	–
Wang et al. (69)	138 (26% ICU)	54.30%	56	Hypertension (31.2%), Cardiovascular disease (14.5%), Diabetes (10.1%), Malignancy (7.2%), Cerebrovascular disease (5.1%), COPD (2.9%), Chronic kidney disease (2.9%), Chronic liver disease (2.9%), HIV infection (1.4%).	ARDS (19.6%), Arrhythmia (16.7%), Shock (8.7%), Acute cardiac injury (7.2%), Acute kidney injury (3.6%).
Zeng et al. (73)	1	100.00%	63	Allergic cough for 5 years. Previous smoking history. Decrease of the cardiac function.	Severe pneumonia, ARDS, fulminant myocarditis, multiple organ dysfunction syndrome.
Hu et al. (74)	1	100.00%	37	Markers of myocardial injury elevated.	Fulminant myocarditis with cardiogenic shock and pulmonary infection.
Liu et al. (77)	137	44.50%	57	Chronic diseases (17.5%), Diabetes (10.2%), Hypertension (9.5%), Cardiovascular disease (7.3%), Chronic obstructive pulmonary disease (1.5%), Malignancy (1.5%). 84% of patients have elevated C-reactive protein levels.	–
Liang et al. (81)	1,600	Cancer patients: 61.10% Non-cancer patients: 57.20%	Cancer patients: 63.1 Non-cancer patients: 48.7	Chronic obstructive pulmonary disease, Diabetes, Hypertension, Coronary heart disease, Cerebrovascular disease, Viral hepatitis type B, Malignant tumor, Chronic kidney disease, and Immunodeficiency. Cancer types: 17 patients with solid cancers including lung cancer and breast cancer), and 1 patient with lymphoma.	–
Zhang et al. (82)	28 (21.4% ICU)	60.70%	65	Diabetes (14.3%), Chronic cardiovascular and cerebrovascular disease (including hypertension and coronary heart disease) (14.3%), Chronic liver disease (including chronic hepatitis B and cirrhosis) (7.1%). Chronic pulmonary disease (including chronic obstructive pulmonary disease and asthma) (3.6%). 82% of patients have elevated C-reactive protein levels. Cancer types: Lung (25%), esophagus (14.3%), Breast (10.7%), Laryngocarcinoma (7.1%), Liver (7.1%), Prostatic (7.1%), Cervical (3.6%), Gastric (3.6%), Colon (3.6%), Rectum (3.6%), Nasopharynx (3.6%), Endometrial (3.6%), Ovarian (3.6%), and Carcinoma of testis (3.6%).	ARDS (28.6%), Pulmonary embolism suspected (7.1%), Acute myocardial infarction (3.6%), Septic shock (3.6%).

*ARDS, acute respiratory distress syndrome*.

### Anti-cancer Drug-Induced Cardiovascular Injury Might Increase COVID-19 Complication Risks

**Figure 4:**
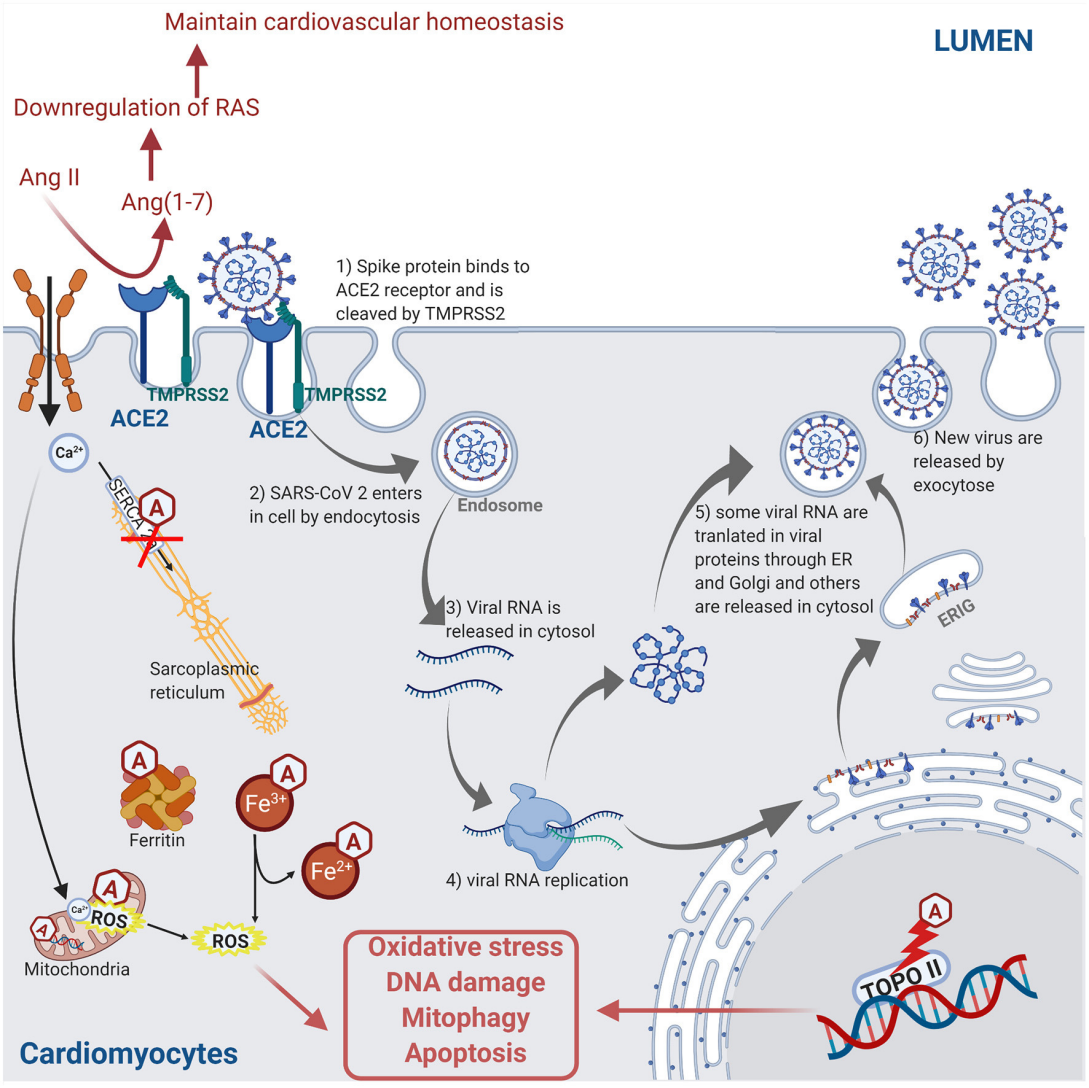
Molecular mechanisms involved during anthracycline (Hexagonal box with “A”) treatment and SARS-CoV-2 infection in cardiomyocyte leading to injury. Anthracycline can bind to nuclear and mitochondrial DNA and interfere with the cell replication process. The TOPO II enzyme can be inhibited by anthracycline, leading to the damaged DNA during cell replication. Anthracycline can also disturb the Ca^2+^ influx into the cell. The disruption of Ca^2+^ homeostasis by anthracycline in the mitochondria leads to an increase of reactive oxygen species (ROS) production and oxidative stress. Moreover, the expression of the Ca^2+^ pump SERCA2a in the sarcoplasmic reticulum is decreased by anthracycline, which induces contractile dysfunction. Anthracycline can also prevent the release of iron from ferritin and facilitate the reduction of anthracycline–Fe^3+^ complexes leading to ROS production. Combined, these anthracycline mechanisms lead to increased ROS production, DNA damage, and contractile dysfunction, which leads to cellular apoptosis. Please refer to Figure 2 legend for a detailed description of the SARS-CoV-2 pathway (figure created with Biorender.com).

Certain anti-cancer drugs can cause severe cardiotoxicity through increased fibrosis, oxidative stress, apoptosis, and necrosis in myocardial cells, thus increasing the risk of cardiomyopathies and heart failure. The anti-cancer drug-induced cardiotoxicity could exacerbate COVID-19-mediated cardiac injury and dysfunction. These drugs include anthracyclines (83, 84), tyrosine kinase inhibitors (85, 86), proteasome inhibitors (87), human epidermal growth factor receptor 2 blocking antibodies (88), and ICIs (89). The intracellular mechanisms involved during anthracycline-induced injury in cardiomyocytes include (a) binding to nuclear and mitochondrial DNA and interfering with replication, (b) inhibiting the TOPO II enzyme leading to the damaged DNA during the cell replication process, (c) disturbing the Ca^2+^ influx into the cell, (d) disruption of Ca^2+^ homeostasis in the mitochondria leading to an increase in reactive oxygen species (ROS) production and oxidative stress, (e) contractile dysfunction due to decreased expression of the Ca^2+^ pump SERCA2a in the sarcoplasmic reticulum, and (f) preventing the release of iron from ferritin and facilitating the reduction of anthracycline–Fe^3+^ complexes leading to ROS production. ROS production, DNA damage, and contractile dysfunction can all lead to cellular apoptosis (3). Figure 4 describes the molecular mechanisms involved during anthracycline treatment-induced injury in cardiomyocytes and link them to the SARS-CoV-2 infection pathway.

A clinical study assessed the outcome of underlying cardiovascular complications in 138 hospitalized COVID-19 patients in Wuhan, China, with underlying cardiac comorbidities: 58% patients were suffering from hypertension, 22% patients had diabetes, and 25% already had a cardiovascular disease (69). Similar findings were published in another report, where 416 hospitalized COVID-19 patients were suffering from co-existing cardiovascular diseases: 60% patient had hypertension, 24% patients suffered from diabetes, 29% patient had coronary heart disease, while 15% patients had suffered chronic heart failure (67). According to the National Health Commission of the People's Republic of China, 35% of COVID-19 patients had hypertension and 17% suffered from coronary heart disease prior to their COVID-19 development (69). These studies indicate that patients with myocardial injury due to underlying cardiovascular diseases are more likely at risk to develop severe complications as a result of SARS-CoV-2 infection (67, 69, 71). Further to this, anti-cancer drugs can trigger late-term cardiovascular adverse effects, such as hypertension, cardiac dysfunction, thromboembolic events, and ischemia heart disease, and these cardiovascular complications are considered high risk factors of developing severe complications during COVID-19 infection in cancer survivors (90).

Cancer patients have a high risk of venous thromboembolism, particularly cancer patients treated with pro-inflammatory drugs and radiotherapy (91, 92). Therapy with anti-cancer drugs has also been associated with venous thromboembolism, which potentially could exacerbate the COVID-19-induced intravascular coagulative damages. These anti-cancer drugs lined with venous thromboembolism adverse effects include anti-VEGF therapies, tyrosine-kinase inhibitors, platinum-based drugs, proteasome inhibitors, hormonal therapy, and immunomodulators (93). Cancer patients initiating or continuing ICI therapy are facing a dilemma during the COVID-19 pandemic. A potential COVID-19 infection is characterized by mild to severe inflammation of the lungs and other organs. Both the ICI-induced immune-related adverse events and the COVID-19-caused inflammation include unrestrained immune and cytokine activation; therefore, ICI therapy could impact the course of COVID-19 and worsen the outcome (94). Cancer patients treated with ICIs are at increased risk of developing myocarditis, and recently, there have been increasing reports that COVID-19 is also associated with the development of myocarditis, with inflammatory cellular infiltrate similar to that seen in ICI-induced myocarditis (95, 96).

### Comorbidity Could Follow a “Two-Hit” Model

Cancer survivors can unfortunately suffer from subclinical myocardial injury due to adverse effect from their anti-cancer therapy (97). A study showed that 65% of cancer survivors treated with anthracycline ended with subclinical cardiac injury (98). Patients treated with anthracycline may suffer from subclinical left ventricle dysfunction, which can possibly lead to clinical cardiomyopathy (99). The cardiac reserve can be defined as “the increase in cardiac function from rest to peak exercise.” In cancer survivors, anthracycline treatment reduces the cardiac reserve, and this parameter can be used as a marker of subclinical cardiac myocardial injury (90). Several reports have concluded that the risk of developing cardiac dysfunction increases over the years after completion of anthracycline cancer treatment, and in the most severe adverse effect cases, heart failure is observed (100–103).

The subclinical cardiac dysfunction post-anti-cancer treatment could potentially serve as a risk factor for COVID-19-induced cardiac injury. Indeed, myocardial injury caused by COVID-19 infection can worsen the outcome consequences in patients with reduced cardiac reserve, leading to the development of severe cardiac complications in these patients. The cardiovascular complications from COVID-19 infection could provoke an acceleration of the myocardial injury resulting from anti-cancer treatment in a “two-hit” manner, as both COVID-19 infection and anti-cancer therapy can lead to myocardial injury (13). As mentioned previously, SARS-CoV-2 downregulates ACE2 expression by binding to ACE2 receptors (47), and ACE2 receptors are essential regulators of cardiac function (49). Therefore, in cancer patients and survivors that have undertaken anti-cancer therapy, a COVID-19 infection increases the potential for cardiac dysfunction due to the SARS-CoV-2-induced downregulation of ACE2 receptor expression. Further to this, the level of the cytokine storm modulator interleukin IL-6 has shown to be increased in more severe cases of COVID-19 compared to mild cases (55, 56). Interleukin IL-6 is produced by tumor cells, and an elevated level of IL-6 has been reported in various cancer types, such as lung cancer (104), renal cell carcinoma (105), and ovarian cancer (106). As a result of this, the elevated levels of IL-6 observed in cancer patients and survivors can exacerbate the effect of a potential COVID-19-mediated cytokine storm in infected patients also through IL-6, which can lead to increased injury in both lung (58) and myocardial tissue (59). On a positive note, a Review by Vivarelli et al. based on preliminary case studies with limited number of patients has hypothesized that cancer patients treated with anti-PD-1 or anti-PD-L1 antibody ICI therapy might potentially benefit from a boosted anti-viral immune response in addition to the T-cell cytotoxic response against cancer (107).

However, more studies are needed to get a better understanding of intracellular cardiac injury mechanisms due to COVID-19 infection and to unravel if these intracellular mechanisms overlap with the anti-cancer drug-induced cardiac injury pathways and mechanisms.

## Conclusion

COVID-19 is a new challenge for cancer patients and survivors treated with cardiotoxic anti-cancer drugs. Cancer patients and survivors are immunocompromised and are therefore more susceptible to develop severe complications resulting from COVID-19 infection. In addition to targeting the respiratory system and causing havoc in the lungs, COVID-19 can induce cardiovascular complications and myocardial injury. Understanding the pathways that lead to cardiac complications during the COVID-19 infection can lead to the development of more targeted and tailored therapy options in at-risk groups, such as cancer patients and survivors, and thus improve the outcome of vulnerable patient groups.

## Author Contributions

All authors contributed to the concept, planning, and writing of this mini-review, and approved the manuscript prior to submission.

## Conflict of Interest

The handling editor is currently organising a Research Topic with one of the authors HM and confirms the absence of any other collaboration. The authors declare that the research was conducted in the absence of any commercial or financial relationships that could be construed as a potential conflict of interest.

## Abbreviations

ACE: angiotensin-converting enzyme
ACE2: angiotensin-converting enzyme 2
Ang(1–7): angiotensin 1–7
Ang II: angiotensin II
ARDS: acute respiratory distress syndrome
BNP: B-type natriuretic peptide
COVID-19: coronavirus disease 2019
cTnI: cardiac troponin I
ER: endoplasmic reticulum
ERIG: endoplasmic reticulum–Golgi intermediate compartment
ICI(s): immune checkpoint inhibitor(s)
ICU: intensive care unit
LVEF: left ventricular ejection fraction
NF-κβ: nuclear factor kappa-light-chain-enhancer
RAS: renin angiotensin system
RIG-I: retinoic acid-inducible gene-I
ROS: reactive oxygen species
SARS-CoV-2: severe acute respiratory syndrome coronavirus 2
TLR: Toll-like receptors
TMPRSS2: type 2 transmembrane serine protease
cTnI: cardiac troponin I
cTnT: cardiac troponin T
WHO: World Health Organization.
